# To Dash or to Dawdle: Verb-Associated Speed of Motion Influences Eye Movements during Spoken Sentence Comprehension

**DOI:** 10.1371/journal.pone.0067187

**Published:** 2013-06-21

**Authors:** Shane Lindsay, Christoph Scheepers, Yuki Kamide

**Affiliations:** 1 School of Psychology, University of Dundee, Dundee, United Kingdom; 2 School of Psychology, University of Glasgow, Glasgow, United Kingdom; University of California San Diego, United States of America

## Abstract

In describing motion events verbs of manner provide information about the speed of agents or objects in those events. We used eye tracking to investigate how inferences about this verb-associated speed of motion would influence the time course of attention to a visual scene that matched an event described in language. Eye movements were recorded as participants heard spoken sentences with verbs that implied a fast (“dash”) or slow (“dawdle”) movement of an agent towards a goal. These sentences were heard whilst participants concurrently looked at scenes depicting the agent and a path which led to the goal object. Our results indicate a mapping of events onto the visual scene consistent with participants mentally simulating the movement of the agent along the path towards the goal: when the verb implies a slow manner of motion, participants look more often and longer along the path to the goal; when the verb implies a fast manner of motion, participants tend to look earlier at the goal and less on the path. These results reveal that event comprehension in the presence of a visual world involves establishing and dynamically updating the locations of entities in response to linguistic descriptions of events.

## Introduction

We use language to refer to events in the world. Studies of language comprehension using the situation or mental model frameworks (e.g., [1]) have helped demonstrate that language users keep track of a wide variety of information about those events, such as their spatial and temporal properties [2], [3]. In recent years, the theory of mental simulation [4] has been used to provide a representational substrate for mental models [5]. The idea is that understanding an event described in language invokes a dynamic multi-modal recreation of the experience of that event, using the same sensorimotor systems that would be involved with the sensorimotor interaction with a real event. Evidence in support of the perceptual simulation theory has come from findings that language comprehension leads to the activation of visual features such as the shape [6] and orientation of objects [7] implied by situations described in discourse. As well as static properties, there is evidence for the activation of more dynamic visual information such as direction of movement [8] and motion of moving objects [9]. For example, when hearing sentences describing the throwing of a baseball, participants were faster to verify whether two pictures matched when the relationship between the pictures was analogous to the motion implied by the sentence [9].

An important aspect of an event is the amount of time it takes to occur. The duration of an event may differ for various reasons, such as the fact that a movement from one location to another can take a long time because the distance is large or the movement is slow. In classic work, Kosslyn, Ball and Reiser [10] showed that scanning time for mental images took longer when there was a longer distance to scan, in the same way that the time it would take to look across a real map would be related to the distance between points on that map. These results are compatible with the theory of mental simulation which posits that simulations are analogues of perception of events in the world. For language processing, this means that comprehension of sentences referring to more ‘time-consuming’ events should lead to more time-consuming simulations.

This idea has been studied in several studies of text comprehension. In children’s narrative comprehension [11], processing times for sentences were longer when agents in a story were described as travelling slowly (walking), compared with travelling quickly (being driven in a car), consistent with the idea that their comprehension of the narrative involved a simulation of the events. Relatedly, Matlock [12] reported a reading study in which processing times for implied speeds of journeys were examined. She used grammatical constructions that Talmy [13] described as co-extension path fictive motion. This kind of fictive motion in English involves the use of a motion verb construction (typically manner neutral) to describe the spatial properties of a path (e.g., “the road *runs through* the valley”) or a linearly extended entity (e.g., “the cord *runs along* the wall”). Matlock found that participants took longer to process sentences where the implied journey was long or slow, and only when fictive motion was described. The speed of the journey was manipulated by different descriptions of the distance of the journey, the difficulty of traversing terrain on that journey, or more explicit descriptions of the speed of the journey (e.g., driving at 100 mph in a Ferrari vs. 40 mph in a VW bus). Further support for the idea that simulations reflect speed of inferred events comes from Yao and Scheepers [14], who found that it took longer to read direct speech quotations silently when the quotations were contextually implied to be uttered by a slow-speaking than by a fast-speaking quoted protagonist, suggesting that participants invoked an analogue mental simulation of the described speech.

Since one of the claims of the simulation approach is that visuo-spatial representations are activated by language comprehension, a natural method to study those representations is through the use of eye tracking. One such study was done by Spivey, Tyler, Richardson, and Young [15], who found that when hearing stories involving events which occur in a particular direction (e.g., on increasingly higher floors of an apartment building) participants’ eye movements mimicked the directionality of the event in the story, suggesting a scanning process akin to constructing a mental model of the described situation. Another eye tracking method that can be used to study simulation in language is the visual world paradigm [16], where spoken language is presented with accompanying scenes. Matlock and Richardson [17] employed this technique in an eye tracking study of sentences with a fictive motion construction. They measured eye movements while participants viewed simple scenes featuring horizontal or vertical paths or linearly extended entities, without any agents or goals. The scenes were accompanied by auditory presentation of sentences with fictive motion constructions describing the spatial properties of those paths and linearly extended entities. These sentences were contrasted with sentences without a motion verb construction, such as “the road is in the valley”. They found longer looking times along the path for the fictive motion sentences, which they argued stemmed from fictive motion constructions invoking a mental simulation of motion which encouraged visual scanning along the paths or linearly extended entities. A follow up study [18] found that time spent looking at paths was influenced by the complexity and difficulty of traversal of that path – additional information that was provided in context sentences heard before scene viewing (e.g., “the valley was flat and smooth” vs. “the valley was full of potholes”). Here participants spent more time inspecting paths in the difficult terrain condition, but only for those sentences with the fictive motion construction.

In the above studies on fictive motion no literal motion of agents was described; while a road may provide a means to support motion events, a cord cannot literally run along a wall. This makes it unclear what they reveal about comprehension of events with moveable agents undergoing actual motion. Instead, rather than revealing the nature of event processing or of figurative language understanding, fictive motion constructions may be seen as a conventionalised means of expressing a spatial property, where verbs are paired with a prepositional phrase which emphasises the linear-path quality of a spatial arrangement (e.g., fictive: “runs along” vs. the non-fictive: “is next to”). Taken together, research on fictive motion supports the idea suggested by Talmy [13] that such constructions invoke a “survey” type (i.e., a bird’s eye view, [19]) emphasizing the spatial property of extension or elongation (“the path goes from the town to the beach”). The results by Matlock and Richardson suggest that these types of spatial representations invoke a dynamic mental simulation which involves scanning along the extent of the featured entity.

**Figure 1:**
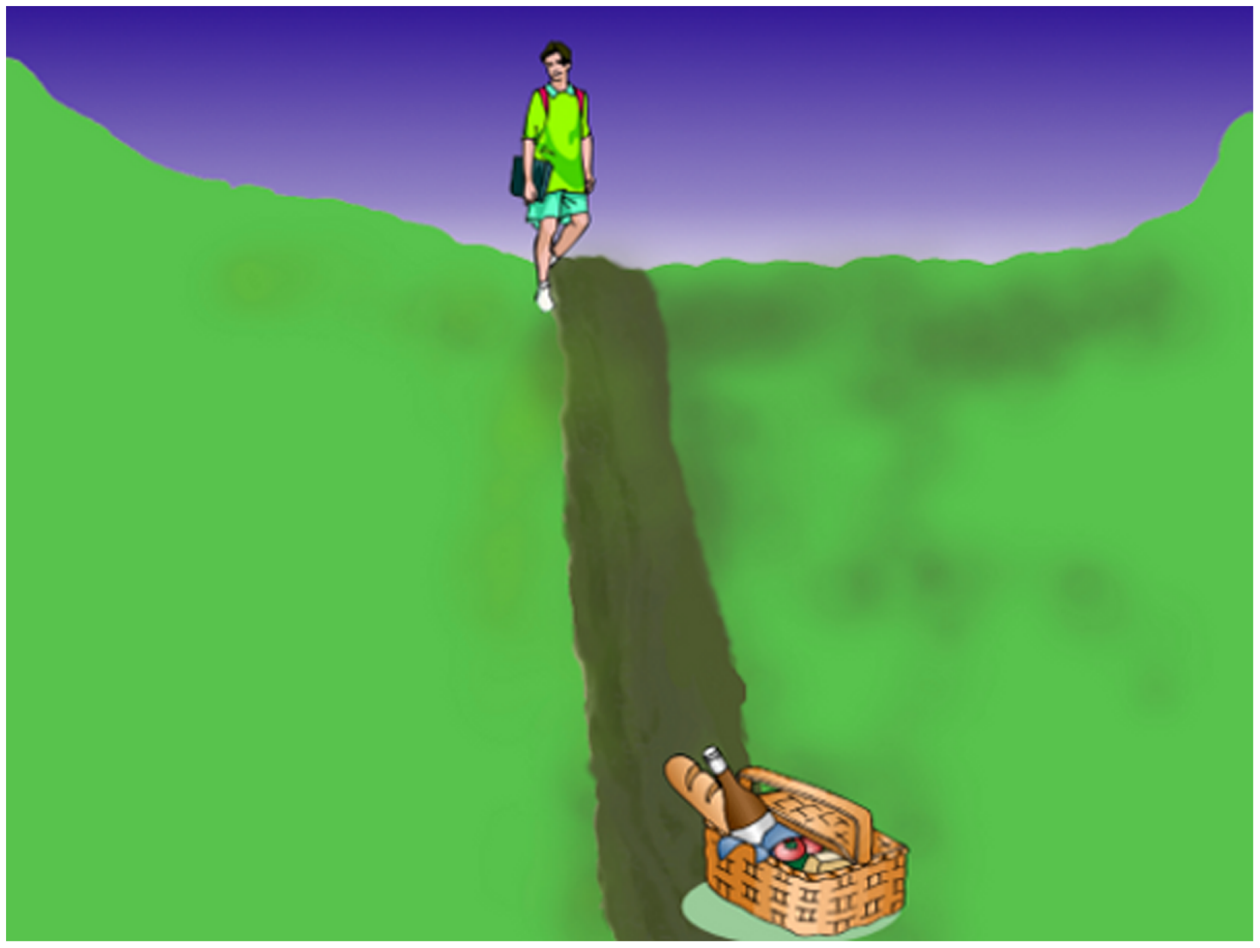
Example scene for the fast/slow sentences: “The student will run/stagger along the trail to the picnic basket”.

In the current work, we also focus on paths, but use an eye tracking visual world paradigm to index the online allocation of visual attention when understanding sentences involving an agent undergoing an actual motion event. One of the advantages of this paradigm is that it allows investigation of the coupling of the visual world and language over time, as language users incrementally build models of events described in language. Earlier work has shown that language-mediated eye movements reflect the mapping of inferred locations of objects featured in a discourse when presenting with static visual scenes [20], with locations being updated online in accordance with the event described by the language. In our experiment below, we presented participants with static scenes depicting an agent, a path and a goal. As they viewed these scenes, they heard a sentence which described an agent undergoing a location change signalled by a verb phrase with a motion verb and path-denoting satellite or prepositional phrase, explicitly signalling the path to a goal. An example scene with accompanying sentences is shown in Figure 1. Crucially, we varied the manner of motion described by the verb. An important aspect denoted by manner of motion is speed, with some manners suggesting fast motion (e.g., “sprint”) and some slow (“crawl”). According to the simulation theory of language comprehension, participants should process linguistically provided information about the speed of a journey in a similar way to how we might perceive that journey in the world. Based on evidence that language users dynamically map and update locations of entities in the visual world in response to language, we expected that when the verb in a sentence suggests a slow movement towards a goal then that journey should be inferred to take longer than if it was a fast movement. As a consequence, eye movements would reveal more looking along the path on which the agent will travel. In contrast, when the verb implies a quick journey, participants should spend less time looking along the path and instead look more and earlier to the goal, as the agents would be projected to arrive at the goal earlier. In addition to eye tracking, we also collected computer mouse movement tracking data [21] for the same stimuli in a separate task, to give us a more direct measure of how participants interpreted the speed at which the agents moved. Using a mouse, participants moved the agent within the scene to the end location described in the sentence. For slow verb sentences we expected it would take longer to move the agent to the goal compared with the fast verbs.

## Methods

### Participants

Forty-three native English speakers without visual or auditory impairments were recruited from the University of Dundee, and received course credits or £4 payment.

### Ethics Statement

The present research was approved by the University of Dundee Research Ethics Committee (Ref: SREC 1103). All participants provided their written informed consent prior to participation.

### Materials and Stimulus Construction

Initially 89 candidate verbs of movement were selected, and we conducted a two-part norming questionnaire with 14 participants. In the first part, each verb was inserted into a sentence in which the agent and goal were replaced with the letters X and Y, and only the verb differed across items. For example: “The X will run along the path to the Y”. This was done to ensure that participants focused on the meaning of the verb and its implied speed of agentive travel, rather than the semantics of the agents and goals. Participants had to rate how quickly the verb implied movement to the goal, with a rating of 1 for “very slowly” and 7 for “very quickly”. Participants then completed a movement suitability questionnaire with the same items, but now had to judge how suitable the verb was for describing a movement of X to Y along a path, where a rating of 1 meant the verb was “very unsuitable” (“a strange way to describe movement from one location to another”) and 7 was “very suitable (“would often use the verb in such a way”). These ratings led to the selection of the 16 fast and 16 slow verbs with the highest or lowest ratings of speed, and suitability ratings above 3.75 (fast: mean speed rating = 6.06, *SD* = 0.49, mean suitability rating = 5.46, *SD* = 0.84; slow: mean speed rating = 2.05, *SD* = 0.40, mean suitability rating = 5.13, *SD* = .65). There was no significant difference in the spoken length of the verbs (*p*>.10), but fast verbs had significantly higher frequency using log10 CELEX lemma frequencies [22] (fast: *M* = 1.28, *SD* = 0.57; slow: *M = *.56, *SD* = .42; *t(32)* = 3.6, *p*<.001).

Thirty-six 1024×768 pixel colour scenes were constructed containing an agent and goal which were selected from a commercially available clip-art database. The background and path were manually created using graphics software and chosen to thematically match the agents and goals (e.g., a canyon landscape for a cowboy and cactus). The paths directly connected the agent and goal with an equal division of horizontal or vertical paths, with the agent appearing close to the middle of one side of the screen, and the goal appearing on the opposite side (see Figure 1 for an example scene and sentence).

Thirty-six sentences (see Table S1 for the full set) were constructed to match the scenes, with each scene having a matching fast and slow verb speed version. Sentences used the same syntax with a future tense marker “will”, the preposition “along”, one of seven possible path nouns (e.g., “track”, “path”, “road”), and a unique agent and goal combination. Each of the 18 fast and 18 slow verbs was paired with two scenes, with sentences counterbalanced across two lists so that a participant saw all scenes but heard each verb only once. Stimuli were recorded by a native speaker of British English at 44,100 Hz. To create each pair of sentences, the verb and the preposition (e.g., “run along”) were cross-spliced using sound editing software from a recording of the fast version of the sentence into a recording of the slow version, to ensure that the sentences were otherwise acoustically identical.

We also included 18 filler scenes with different verbs to the experimental items, which involved an equal split of either omission of the path from the scene and accompanying sentence (e.g., “The sheep will travel to the tractor”), removal of the goal from the scene and sentence (e.g., “The waiter will walk along the path”), or had a non-movement verb without a path in the scene or sentence (e.g., “The sailor will dream about the windmill”).

Finally, we conducted an additional norming study on Amazon Mechanical Turk in which 24 participants took part. Experimental and filler sentence-picture combinations appeared in a random order and participants had to rate the suitability of each agent-verb combination (“how suitable do you think the verb is for combining with the agent in describing movement of that agent along a path to another location”) on a 7-point scale (1 for very unsuitable, 7 for very suitable). In the instructions, it was stressed that ratings should not be based on how suitable the path or goal was. Along with the experimental items, we included the 18 fillers, and adapted their verbs to make them less suitable (e.g., “The tiger will puncture to the house”), ensuring a wide range of responses. Ratings for suitability were slightly higher for the fast verb sentences (fast: *M = *4.7, *SD* = 0.9; slow: *M* = 4.2, *SD* = 1.2; *t1*(22) = 3.6, *p*<.001; *t2*(71) = 2.01, *p* = .04).

### Procedure

Participants’ eye movements were recorded using a head mounted SR Research Eyelink II tracker sampling at 500 Hz. Viewing was binocular but eye movements were recorded from one eye. Scenes were viewed at a distance of approximately 60 cm on a 120 Hz 21″ CRT monitor and sentences were played at a conversational level in mono across a pair of speakers positioned on either side of the computer monitor. Participants were instructed that they were going to view some scenes with accompanying sentences describing an event, and their task was to “listen carefully to each sentence while looking at the screen, and try to understand what will happen in each scene”. Scenes were presented for 1000 ms before onset of the spoken sentences to allow examination of the display before hearing the sentences. The trial finished 8000 ms after sentence onset. Mean sentence length was 4188 ms (*SD* = 354 ms). Trial order was randomised per participant. In between each trial a centrally located dot was presented which participants had to fixate upon before the trial started, to allow for an automatic drift correction. After every 8^th^ trial the eye tracker was recalibrated using a 9-point fixation stimulus, which took approximately 20 s. Four practice trials were presented to allow participants to become familiar with the procedure. The eye tracking portion of the experiment took approximately 25 minutes.

After a short break, the same participants performed a mouse tracking task with the same stimuli, with a different randomised order of trials per participant. Participants were given the following instructions: “Your task is to listen carefully and understand the event being described in the scene, and then after you hear the tone you should use the mouse to drag the agent to where the sentence describes it will move to”. The tone appeared 1000 ms after the sentence ended, which co-occurred with the appearance of a cursor in the middle of the screen. Participants used their self-reported dominant hand to “drag and drop” the agent using the left mouse button. There was no time limit for responding. Trials ended after the release of the mouse button, giving participants only one opportunity to move the agent. Only the agent could be moved. For those sentences in which they did not think the agent moved at all (which was implied in some of the filler items) they were instructed to press the space bar to move on to the next trial. Four practice trials were given. This part of the experiment took approximately 15 minutes.

## Results

Contrary to the chronological order, we report the mouse tracking data first, as we expected that without an effect in the mouse tracking task, differences in eye movements would be minimal.

### Mouse Movement Tracking Analysis

We used the recording of the time between the first click on the mouse button (“picking up” the agent) and its release (“dropping” the agent at the end destination) as a measure of how quickly participants interpreted the speed of movement in the scenes.

**Figure 2:**
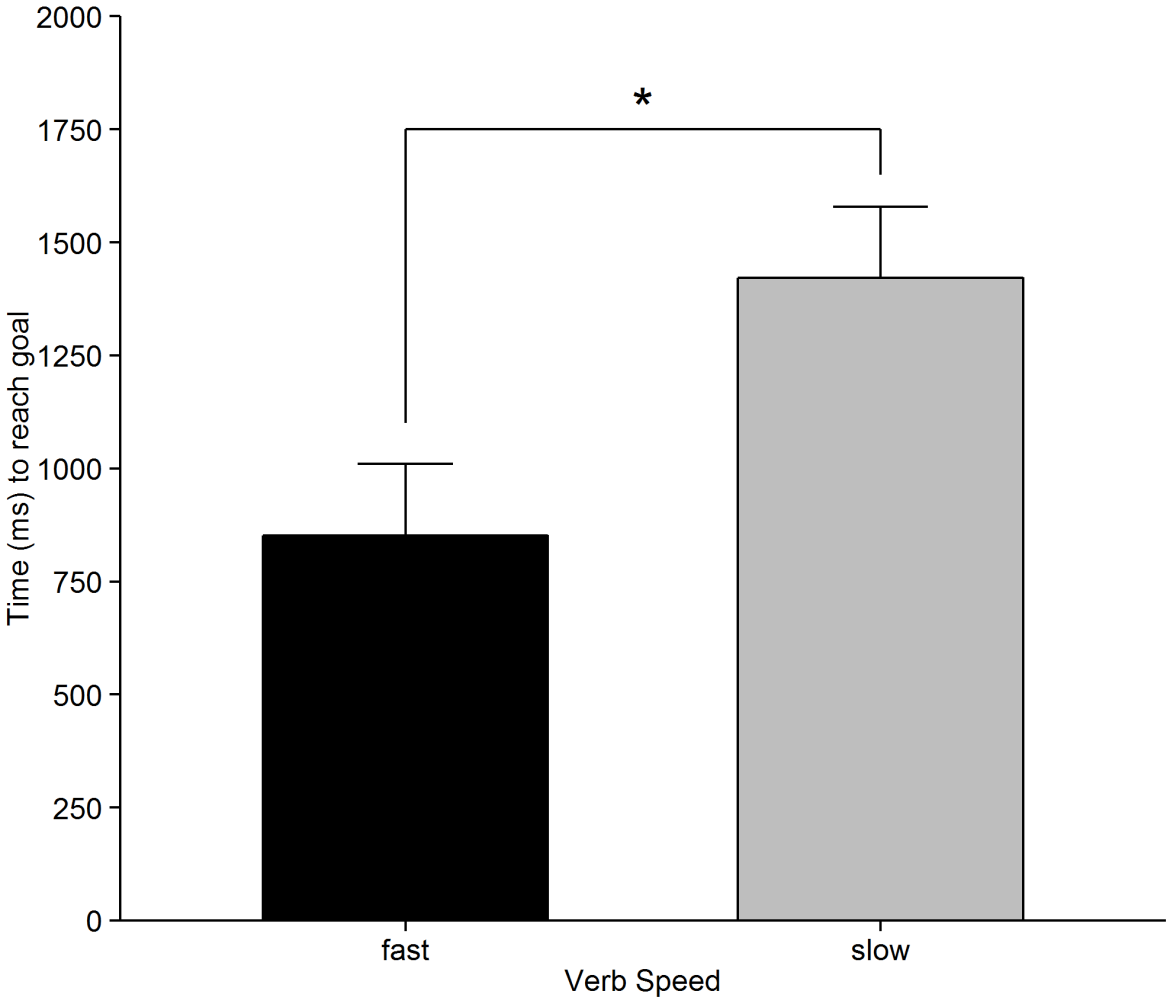
Mean movements times for the movement tracking task. Error bars indicate within-participant 95% confidence intervals [35].

Results were analysed using *Generalized Estimating Equations* (GEE) [23], [24] using a *Gaussian* distribution and *identity* link function, with Verb Speed (fast vs, slow) as a categorical predictor and both lexical frequency of the verb (CELEX log10 frequencies) and agent-verb suitability (Mechanical Turk ratings per item) as continuous predictors. The frequency and suitability covariates were included to examine whether effects of Verb Speed occur above and beyond potential influences of these confound variables on processing time; we will not report any significant effects of these covariates as they are not of theoretical interest. Two types of analyses were performed: one treating the three predictors within-participants variables (reported as 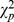) and one treating the predictors as within-items variables (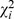). In each case, an exchangeable covariance structure was assumed for repeated measurements. Participants took significantly longer to move the agent to the end location for the slow Verb Speed condition (*M* = 1422 ms, *SD* = 1041 ms) compared with the fast Verb Speed condition (*M* = 852 ms, *SD* = 384 ms) [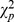
(1) = 96.01, *p*<.001; 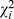
(1) = 120.93, *p*<.001]. These results are shown in Figure 2. The effects were not due to the amount of distance travelled, as there was no significant difference between conditions in terms of number of pixels moved from start to end location (*p*s >.05). The mouse tracking data corroborate the results from the movement-speed ratings by showing that, when embedded in meaningful sentences with accompanying visual scenes, speed-associated verb information influences the interpretation of how long it takes an agent to reach the goal (note that speed was actually not explicitly mentioned in the mouse tracking instructions). Indeed, there was a very strong correlation between the speed ratings in the norming study and the mouse tracking data (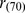 = .87, *p*<.001). In the following eye tracking analysis we focused on online incremental interpretation as the sentence was unfolding.

### Eye tracking: Looking Time Analysis

To examine eye movements, three spatial Regions of Interest (ROI) per scene were assigned manually using the SR Research Data Viewer software. This was done by constructing rectangles which contained boundaries of the Agent and Goal, and a four-sided polygon which enclosed the Path. In cases where the Path ROI overlapped with either the Agent or the Goal ROI, fixations on those ‘ambiguous’ areas were counted as being on the Agent or Goal, respectively.

**Figure 3:**
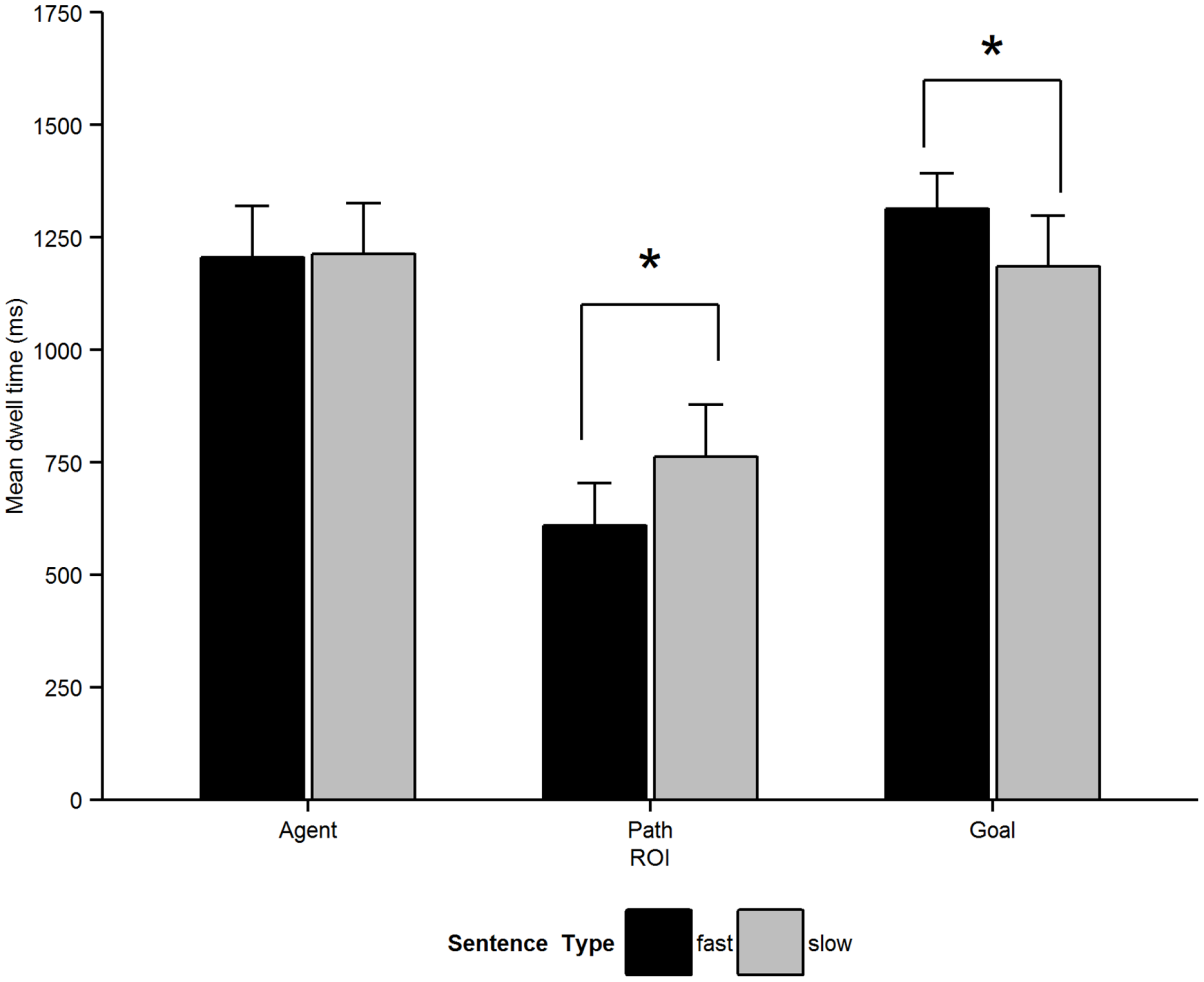
Mean total dwell time (total looking time per trail from verb onset to sentence offset) for the Agent, Path and Goal ROI. Error bars indicate 95% within-participants confidence intervals**.**

We first analysed total dwell time, which was the summed total duration of fixations within a ROI across a time window. As we expected shifts in visual attention to be modulated by verb semantics we examined the time window from verb onset to the end of the sentence. The mean duration of the time windows were 3150 ms for the fast verb condition and 3177 ms for the slow verb condition. Dwell time results are shown in Figure 3. Analyses employed the same GEE modelling approach as described in the mouse tracking section, but with separate models for looks to the Agent, Path and Goal ROIs. Also, we now used a *gamma* distribution and *log* link function to better account for the fact that the dwelling time distributions were positively skewed. As predicted, participants spent significantly longer looking at the Path for slow verb sentences compared with fast verb sentences [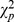 (1) = 9.79, *p*<.001; 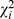
(1) = 4.64, *p = *.031]. In contrast, there was longer dwelling on the Goal ROI for fast verb sentences, significant by-participants and marginal by-items [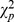
(1) = 5.51, *p = *.019; 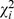
(1) = 3.53, *p = *.06]. There were no significant differences in dwell times for the Agent ROI (*p*s >.4).

### Eye tracking: Proportions of Fixations over Time Analysis

Complementing our analysis of dwell time, we analysed proportions of fixations over time. While this measure is typically correlated with dwell time (more fixations should lead to longer overall looking times), the present analysis has the advantage of revealing fine-grained changes in attention to different ROIs over time.

**Figure 4:**
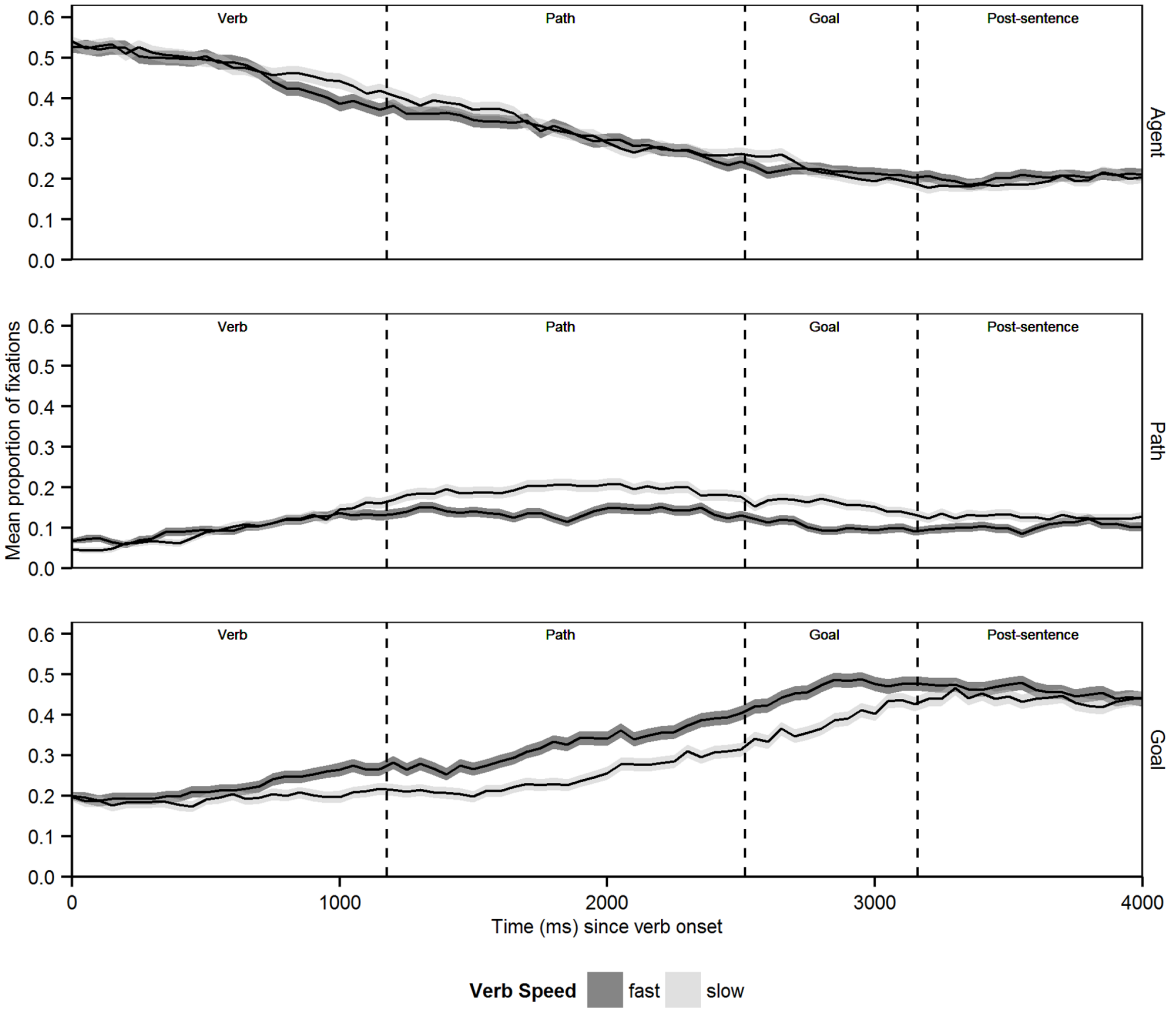
Mean proportions of fixations for Agent, Path and Goal regions, where 0 ms = verb onset. Shaded area around mean line indicates 1± std. error from the mean.

Mean proportions of fixations over time (separately for each ROI) are shown in Figure 4, with aggregation of data by 50 ms time bins. In line with the dwell-time results, Figure 4 shows that looks to the Agent ROI were not affected much by experimental condition, therefore further analyses focused on the theoretically most relevant Goal and Path ROIs only. Descriptively, Figure 4 suggests that the cross-condition difference in dwell time for the Path ROI is globally attributable to a difference in overall likelihood of looks, with a higher ‘peak’ of the curve in the slow than in the fast Verb Speed condition. Particularly noticeable for the Goal ROI is an early ’anticipatory’ bias towards this region, shown by curves rising well in advance of the goal noun being mentioned at the end of the sentence. This was not unexpected, as the nature of the scenes and sentences used made the Goal easy to predict, and these anticipatory effects have been previously documented in visual world experiments [25]. What was also apparent for the Goal ROI was a cross-condition difference in these anticipatory looks. The bottom panel of Figure 4 suggests that this cross-condition difference can be characterised as a difference in timing of visual attention: relative to the slow Verb Speed condition, the upwards curve for the fast Verb Speed condition appears to be shifted more to the left on the time dimension.

To qualify these observations more formally we divided the considered time period into four smaller time windows of interest (on a by-trial basis) based on critical linguistic information available. These four windows are shown in Figure 4: the *Verb* window (mean length = 1159 ms; from onset of the verb to onset of the path noun; e.g., “run_along_the_”; “_” indicates a pause in the speech streams); the *Path* window (mean length = 1340 ms; from onset of the prepositional noun to the onset of the goal noun; e.g., “trail_to_the_”); the *Goal* window (mean length = 647 ms; from goal noun onset to the end of sentence, e.g., “picnic basket.”); and the *Post-sentence* window (from end of sentence until 1000 ms later, to establish the persistence of any verb-associated differences in the absence of linguistic inputs).

The trial-level data per ROI (*Path*, *Goal*) and time window (*Verb*, *Path*, *Goal*, and *Post-sentence*) were statistically modelled using *GEE*. Given that responses at trial by time-bin level were dichotomous (a fixation on a given ROI either occurs or not), we specified a *binomial* distribution and *logit* link function in these analyses. Two types of analyses were performed per ROI and time window of interest: the first treated Verb Speed (fast vs. slow) and Time (continuous predictor referring to the 50 ms time bins per window) as within-participants predictors (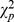) and the second treated the two variables as within-items predictors (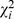). Effects of Verb Speed refer to condition-dependent differences in the overall likelihood of looking within a time window; the slope of the continuous Time variable indicates changes in proportions of looks over time, with the coefficient indicating an increase (positive slope), decrease (negative slope) or no change at all (slope around zero). To account for the fact that time series data tend to be more correlated for adjacent time bins than for time bins that are further apart, a *first order auto-regressive* (AR1) covariance structure was assumed. The results from these analyses are shown in Table 1 (looks to the Path ROI) and Table 2 (looks to the Goal ROI) and will be summarised in the following sections.

**Table 1:** Binary logistic GEE results for the Path ROI (per time window of interest).

	By-participants (N = 43)							By-items (N = 36)			
Window	Effect	Est.	*SE*	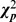	*p*	Δ*QIC*	Est.	*SE*	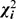	*p*	Δ*QIC*
Verb	Speed	+.306	.178	2.67	.10		+.307	.187	2.64	.11	
	Time	+.058	.008	15.18	.001	+37.5	+.062	.010	24.11	.001	−48.3
	S×T	−.017	.010	2.60	.11		−.016	.011	2.41	.12	
Path	Speed	−.500	.132	10.33	.001		−.501	.116	12.48	.001	
	Time	−.002	.007	0.04	.85	−108.7	−.002	.005	0.09	.76	−200.0
	S×T	+.005	.009	0.36	.55		+.006	.006	1.01	.31	
Goal	Speed	−.498	.166	5.92	.02		−.502	.146	8.87	.003	
	Time	−.029	.014	4.61	.04	−113.2	−.030	.012	5.15	.03	−193.4
	S×T	+.004	.019	0.05	.83		+.005	.012	0.15	.70	
Post-sent.	Speed	−.364	.189	3.35	.07		−.362	.142	5.67	.02	
	Time	+.004	.009	1.56	.21	−58.6	+.004	.002	4.23	.04	−183.5
	S×T	+.013	.012	1.28	.26		+.013	.008	2.26	.13	

Table shows GEE modelling results for occurrences of looks to the Path ROI as a function of Verb Speed (fast vs. slow) and Time (continuous predictor referring to the 50 ms time-bins per window). Shown are the by-participant/by-item GEE parameter estimates and corresponding *SE*s (both in logit units) along with *generalised score chi square* statistics and related *p*-values (at *df* = 1). As for interactions (S×T), a positive parameter estimate indicates a more positive slope for the Time predictor in the fast than in the slow Verb Speed condition. ΔQIC refers to the goodness of fit of the *Verb Speed×Time* model in relation to a competitor *Agent-Verb Suitability*×*Lexical Frequency*×*Time* model (negative ΔQICs indicate superior fit of the *Verb Speed*×*Time* model).

**Table 2:** Binary logistic GEE results for the Goal ROI (per time window of interest).

	By-participants (N = 43)							By-items (N = 36)			
Window	Effect	Est.	*SE*	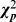	*p*	Δ*QIC*	Est.	*SE*	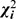	*p*	Δ*QIC*
Verb	Speed	−.017	.138	0.02	.90		−.021	.132	0.03	.87	
	Time	+.009	.004	5.50	.02	−34.9	+.010	.004	6.78	.01	−176.7
	S×T	+.017	.008	3.99	.05		+.018	.006	5.64	.02	
Path	Speed	+.424	.118	10.37	.001		+.422	.110	10.48	.001	
	Time	+.033	.005	23.90	.001	−192.3	+.036	.005	25.87	.001	−325.1
	S×T	−.004	.006	0.35	.55		−.004	.006	0.44	.52	
Goal	Speed	+.380	.133	6.97	.01		+.377	.104	9.32	.002	
	Time	+.029	.010	7.91	.005	−66.5	+.030	.012	7.24	.01	−91.1
	S×T	−.018	.012	2.09	.15		−.017	.011	1.92	.17	
Post-sent.	Speed	+.130	.092	1.91	.17		+.128	.098	1.63	.20	
	Time	−.006	.004	3.38	.07	−2.9	−.007	.003	3.98	.05	−93.8
	S×T	−.001	.007	0.03	.88		−.001	.006	0.03	.87	

Table shows GEE modelling results for occurrences of looks to the Goal ROI as a function of Verb Speed (fast vs. slow) and Time (continuous predictor referring to the 50 ms time-bins per window). Shown are the by-participant/by-item GEE parameter estimates and corresponding *SE*s (both in logit units) along with *generalised score chi square* statistics and related *p*-values (at *df* = 1). As for interactions (S×T), a positive parameter estimate indicates a more positive slope for the Time predictor in the fast than in the slow Verb Speed condition. ΔQIC refers to the goodness of fit of the *Verb Speed*×*Time* model in relation to a competitor *Agent-Verb Suitability*×*Lexical Frequency*×*Time* model (negative ΔQICs indicate superior fit of the *Verb Speed*×*Time* model).

Although it would have been desirable to also include the verb frequency and agent-verb suitability covariates in these models (analogous to the previous mouse tracking and dwell time analyses), this turned out to be computationally less feasible with the present data. Test runs using a full four-factorial design (Verb Speed×Agent-Verb Suitability×Lexical Frequency×Time) yielded an estimated processing time requirement of about 190 hours across all ROIs and time windows of interest. Moreover, there was a risk that the four-factorial design would overfit the data or even fail to converge in some instances (note that parameters are harder to estimate with binary data).

Instead, we ran additional binary logistic GEE analyses per ROI and time window of interest (using the same distribution, link function, and covariance settings as before), but replacing the Verb Speed×Time design with an Agent-Verb Suitability×Lexical Frequency×Time design. Hence, the new “competitor models” comprised Time, Lexical Frequency, and Agent-Verb Suitability as continuous predictors in a full-factorial model. We then compared the goodness of fit of the competitor models (Agent-Verb Suitability×Lexical Frequency×Time) with the goodness of fit of the original models (Verb Speed×Time) for each ROI and time window of interest. The measure used for this purpose was the Quasi-likelihood Information Criterion (QIC) metric specifically developed to quantify goodness of fit in GEE [26]. The results of these model comparisons are shown in the Δ*QIC* columns in Tables 1 and 2, where Δ*QIC* refers to the QIC-difference between the original model and the competitor model. Whenever Δ*QIC* is negative, the original Verb Speed×Time model obtained a better fit of the data than the alternative Agent-Verb Suitability×Lexical Frequency×Time model, indicating that potential Verb Speed effects were not fully attributable to the covariates.

#### Proportions of looks to the Path ROI

Within the *Verb* window, proportions of looks to the Path ROI grew about equally positively in each Verb Speed condition, as reflected in a significant main effect of Time (associated with a positive slope coefficient), and no main effect of, or interaction with, Verb Speed. Within the *Path* window no reliable effect or interaction with Time was detected; instead, there was a significant main effect of Verb Speed such that looks to the Path ROI were generally more likely in the slow than in the fast condition) [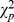
(1) = 10.33, *p = *.001; 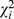
(1) = 12.48, *p*<.001]. For the *Goal* window there was a main effect of Verb Speed (more looks to the Path ROI in the slow than in the fast verb condition, as before) [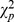
(1) = 5.92, *p = *.015; 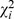
(1) = 8.87, *p = *.003], and a main effect of Time which was associated with a negative slope coefficient (meaning decreasing probabilities of looks to the Path ROI over subsequent time-bins) [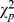
(1) = 4.61, *p = *.032; 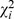
(1) = 5.15, *p = *.023]. The same pattern of results also emerged in the *Post-sentence* window, but the corresponding effects were reliable by items only [*Speed*: 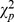
(1) = 3.35, *p = *0.067; 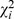
(1) = 5.67, *p = *.017; *Time*: 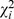
(1) = 1.56, *p = *.211; 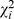
(1) = 4.23, *p = *.040].

The Δ*QIC* values in Table 1 reveal generally superior fits of the Verb Speed×Time model in comparison to the alternative Agent-Verb Suitability×Lexical Frequency×Time model, especially in time windows with significant Verb Speed effects.

#### Proportions of looks to the Goal ROI

For the *Verb* window, a reliable main effect of Time emerged [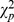
(1) = 5.50, *p = *.019; 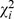
(1) = 6.78, *p = *.009] which was further modulated by a significant Verb Speed×Time interaction [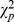
(1) = 3.99, *p = *.046; 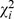
(1) = 5.64, *p = *.018]; the main effect was due to a reliably positive slope coefficient for the Time variable (meaning increasing probabilities of looks to the Goal over time) and the interaction was due to the fact that this slope coefficient was significantly more positive (steeper slope) in the fast than in the slow Verb Speed condition. This finding corroborates the pattern in Figure 4 which indicates a tendency to look earlier at the Goal ROI in the fast Verb Speed condition, and helps provide a more nuanced account for the longer dwell times on the Goal ROI: participants started to look earlier at the Goal for the fast verb sentences, and this led to longer dwell times overall.

Results for the two time windows that follow (*Path* and *Goal*) were qualitatively the same, showing a main effect of Time (due to reliably positive slope coefficients overall) and a main effect of Verb Speed (due to proportions of looks to the Goal ROI being higher in the fast than in the slow Verb Speed condition), but no interaction [*Path Time Window* - *Speed*: 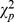
(1) = 10.37, *p = *.001; 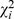
(1) = 10.48, *p = *.01; *Path Time Window - Time*: 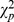
(1) = 23.90, *p<*.001; 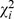
(1) = 25.87, *p<*.001; *Goal Time Window - Speed*: 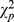
(1) = 6.97, *p = *.008; 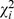
(1) = 9.32, *p = *.002; *Goal Time Window - Time*: 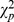
(1) = 7.91, *p = *.005; 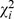
(1) = 7.24, *p = *.007]. In the final time window of interest (*Post-sentence;* from end of sentence until 1000 ms later) the main effect of Verb Speed disappeared, implying that the two curves rose to roughly the same peak proportion of looks to the Goal ROI at maximum time. The only close to reliable effect in this time window was a main effect of Time with negative coefficient [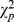
(1) = 3.38, *p = *.066; 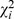
(1) = 3.98, *p = *.046].

Again, the Δ*QIC* values in Table 2 were consistently negative, indicating that Verb Speed×Time yielded a better account of the data than Agent-Verb Suitability×Lexical Frequency×Time.

### Item Correlation Analysis

To further demonstrate that our effects were driven by the speed of journey implied by the verb (rather than some other factor), we correlated the movement times (in ms) from the mouse tracking task with the dwell time data from the eye tracking task on a by-item basis. There was highly reliable positive correlation between the mean amount of time dwelling on the Path (eye tracking) and how long it took participants to move the agent to the goal location (mouse tracking): *r*_(70)_ = .54, *p*<.001). In contrast, there was a non-significant negative correlation between dwelling times on the Goal and the mouse-movement times ( = −.16, *p* = .19). We also correlated mean gaze proportions (from verb onset to sentence end) with the time it took participants to move the agent to the goal in the mouse-tracking task. Again, we found a significant positive correlation between the mouse-tracking data and gaze proportions on the Path (*r*_(70)_ = .43, *p*<.001), and a significant negative correlation between the mouse-tracking data and gaze proportions on the Goal (*r*_(70)_ = −.36, *p*<.01). No such correlations were obtained between eye-tracking data on the agent ROI and the mouse-tracking data (all *p*s >.1). Unsurprisingly, given that the offline speed ratings were highly correlated with the mouse-tracking data, correlations between speed ratings and eye tracking measures gave a similar pattern of results. In summary, verb-related speed of motion influenced participants’ interpretation of how fast the agent would move to the goal, as reflected in the corresponding mouse-tracking data and in participants’ eye movement behaviour.

## Discussion

Taken together, the present results show that dynamic information associated with the inferred speed of a linguistically described motion event influences visual attention and motor execution in a way consistent with simulation-based accounts of language comprehension [5], [27]. In other words, our results support the idea that participants simulate the movement of the agent along the path to the goal. For sentences with verbs that imply a slow motion towards a goal, we expected that participants would allocate greater visual attention along the path of travel to that goal. This was confirmed by our eye tracking results which showed that slow verb sentences led to more time looking along the path and more looks to the path. For sentences with verbs implying faster movement towards the goal, we found that participants were actually faster to look at the goal than for sentences with verbs implying slow movement towards the goal. Because of this, they also spent more time in total on the goal region in the fast verb condition. Thus, if the verb implies fast movement towards the goal, listeners spend less time looking at the path region and more quickly shift their visual attention towards the goal region (in line with a simulated fast movement); conversely, if the verb implies a slow movement towards the goal, listeners are less quick to attend to the goal and spend more time looking at the path region instead (in line with a simulated slow movement). These speed-associated differences in interpretation were confirmed in our mouse tracking task which showed that fast verbs led to faster movements of the agent to the goal than the slow verbs.

In interpreting these findings it is important to clarify how simulation contributes to language comprehension in the visual world paradigm. One perspective on simulation in language comprehension is that it provides a tool for enriching inferences about the visual-spatial properties of situations, the kinematics of motor actions, and emotional and introspective states. This is beneficial as the semantic specification provided via language is schematic [28]. The scenes viewed in the visual world paradigm provide additional information to help flesh out skeletal language semantics, allowing for a richer specification of the visual-spatial properties of the described event than provided by language alone. Our assumption is that attention in the visual world paradigm can index how participants track the locations and changes in location of objects as indicated by unfolding linguistic information. Our use of the term “simulation” is a description of the process of *mapping out* these dynamic visuo-spatial inferences onto a static visual scene. In keeping with the theory of simulation, this may involve some of the same sensorimotor systems used in apprehending such events in the real world. Furthermore, the visual world context may provide a way of cognitive out-sourcing, where an internal mental model which combines linguistic and visual world information is dynamically mapped out onto the visual world, using the latter as an external representational system [29].

In this study we manipulated the speed of the journey implied in an event by use of different manner of motion verbs. Potentially there are other ways to manipulate speed of journey, such as changing the difficulty of the path [18], the intentions of the agent [11], the mode of transport (e.g., by car vs. on foot, [11]), or the implied speed of the agent (a racing boat vs. a tug boat). In principle, if the construction of a mental model for a movement event involves a multimodal simulation, then we should find that visual attention to a depiction of that event should be modulated by the mental model consistent with an “acting out” of that event, regardless of whether crucial information for that simulation process stems from the verb, (visible) situational affordances, or linguistic context.

While our results depend on the use of a visuo-spatial context that promotes activation of perceptual representations, other studies have shown evidence for simulation of event speed in the absence of a visual world, such as in the rate of talking in quotations of direct speech [14], [30]. However, there may be limitations in how simulation based accounts can straightforwardly explain comprehension of temporality in language. Based on the findings that sentences describing longer events can take longer to process, Coll-Florit and Gennari [31] argued that processing time differences may occur because longer events are associated with greater complexity and therefore lead to greater number of inferences. While these results are not entirely incompatible with simulation approaches, for long and complex events a simple analogue relationship between the event described and a simulation of that event is not tenable. For example, it is hard to conceive how one could simulate the running of a whole marathon. Instead, simulation would appear to need scaling and temporal compression, where the time involved in a simulation of an event should have a proportional relationship to the length of an event, but simulations are compressed to allow the generation of inferences appropriate to the discourse context in the time available for discourse comprehension. This compression may involve particular focus on start states, intermediate states, end states and the transitions between them.

Along with manner of motion, verbs can provide information about paths. Our results indicate that language semantics modulates attention to depicted paths when the language explicitly describes agents travelling on them. In future work we are exploring how path information that is not visually depicted but implied by the verb (e.g., “jump” being associated with an upwards path) influences visual attention. It remains to be seen whether similar results could be found without explicit naming of the path and its depiction in the visual world. Since fixations in the visual world are primarily drawn to denoted objects rather than blank areas of the screen, the present naming and depiction of the path could have promoted looks to the region in between the agent and the goal which otherwise might not have occurred. Along with focusing attention on the path and the journey along it, mentioning the path in the sentence may also provide sufficient time for participants to incrementally and rapidly update the position of the agent as they hear the sentence. We also suspect that the results may have been influenced by the richness of other types of manner information associated with the verbs. Informal examination of the verbs suggests that the slow verbs (such as “stagger”) were generally more manner rich than the fast verbs, which included verbs like “hurry” and “rush”. One prediction from the application of the simulation theory to visual world language comprehension is that participants map visual inferences about characteristic manners of motion onto the visual world. If so, then manner rich verbs such as “stagger” could enhance attention to the agent and/or the path along which the agent moves, compared with verbs like “go” which do not focus on the manner of movement. Note that we found no differences in visual attention to the agent as a function of verb-related speed of motion. However, if participants dynamically update the inferred location of the agent as they hear the sentence, then inferences about manner would be projected onto the updated location of the entity, such as its position on the path or in reaching the goal, rather than its starting position visible on screen. As speed of motion is at least partly determined by manner of motion (i.e., “staggering” is slow because it is a cumbersome and stilted movement) it is difficult to disentangle their individual contributions on the basis of the present data. This question awaits future clarification.

We have recently come across a similar study to ours which also manipulated the implied **speed** of verbs using a visual word world paradigm [32]. They used sentences where no path was mentioned (e.g. “the lion ambled/dashed to the balloon”), but with scenes that included a path. Across two experiments, they found that dwelling times on the goal were longer for slow verb sentences than for fast verb sentences, a result opposite to our findings (however, note that they did not report any data for the path region). Dwell time was calculated from the start of the sentence to approximately 1500 ms after the end of the sentence (compared with verb onset to sentence end in our study). One explanation for the discrepancy in results is found in the descriptive data they present for proportions of looks over time, which should be highly correlated with dwell time. Like in our results, there appeared to be a tendency to look at the goal *earlier* in the fast verb condition, but in contrast to our study, there was a much steeper decline in goal-directed looks following the end of fast-verb sentences, which suggests that their dwell time results could have been more strongly influenced by processes that were initiated *after* hearing the sentence. Note that our sentences were actually much longer (due to an additional prepositional phrase denoting the path), which is likely to allow more time to construct an online spatial representation of the event. In contrast, the shorter sentences in [32] may have caused participants to delay this spatial simulation process until after the end of the sentence.

From the mouse tracking results we found that participants moved the agent consistent with the speed implied by the verb. This manipulation differs from experiments involving sentence comprehension where participants respond to sentences using a motor movement which is indirectly related to the event described in the language [33], [34]. The authors of these studies have argued that activation of motor information is an automatic by-product of language comprehension through a simulation process. While the mouse-tracking data may have reflected such perceptual simulation processes as well, we are more cautious about making such a strong claim here. Instead, the mouse tracking data largely confirm the results of the speed-rating norming study. In the mouse-tracking instructions, we only asked participants to move the described agent to the described goal, but participants may have interpreted this as being about verb-related manner of motion as well. Individual mouse-movement patterns suggest that this might be the case for at least some participants. For example, given the verb “limp” several participants moved the agent up and down consistent with a limping movement. In the eye tracking study (which always preceded the mouse-tracking task) such demand characteristics are less likely to explain the results because the fixation data were collected online during sentence processing, rather than offline as in the mouse-tracking task. Eye movements are rapid, ballistic motor responses and it appears implausible that participants ‘acted out’ the manner of motion implied by the verb using their eyes.

To conclude, our results indicate that the verb-related speed at which an event would take place influences the way in which listeners shift their visual attention online as language unfolds. These results support the idea that language comprehension involves dynamic mental simulation of linguistically described situations. Challenges remain in clarifying how online methods such as visual world eye tracking reveal the integration of simulation-driven mental representations with their referents in the visual world, and how these processes operate in conjunction with other non-simulation based language comprehension mechanisms. In the meanwhile, our results contribute to a better understanding of the links between the processing of motion events in the language domain and consequential attention shifts in the visual domain.

## Supporting Information

Table S1 Experimental sentences used in experiment.
(DOCX)
