# Supplementary material for: To Dash or to Dawdle: Verb-Associated Speed of Motion Influences Eye Movements during Spoken Sentence Comprehension
Source: PLoS One. 2013 Jun 21;8(6):e67187. doi: 10.1371/journal.pone.0067187 (PMC3689699; doi:10.1371/journal.pone.0067187)
Supplement: Table S1 — Experimental sentences used in experiment. (DOCX) [file pone.0067187.s001.docx]

# Table S1

Experimental sentences used in experiment.

| Sentence (*fast verb/slow verb*) |
| --- |
| \| The jeep will *race/amble* along the road to the shop. \| \| --- \| \| The repairman will *race/hobble* along the road to the car. \| \| The hiker will *sprint/mope* along the trail to the cottage. \| \| The horse will *gallop/hobble* along the path to the barn. \| \| The van will *hurry/shuffle* along the road to the mansion. \| \| The dingy will *speed/drift* along the river to the tree. \| \| The boat will *rush/dawdle* along the river to the bridge. \| \| The truck will *dart/lumber* along the track to the temple. \| \| The cowboy will *fly/limp* along the trail to the cactus. \| \| The doctor will *dash/shuffle* along the street to the ambulance. \| \| The cleaner will *hurtle/stagger* along the street to the lamp post. \| \| The fireman will *jet/saunter* along the street to the bench. \| \| The dog will *bolt/sneak* along the street to the bin. \| \| The corgi will *dart/trudge* along the street to the table. \| \| The cat will *gallop/inch* along the street to the tree. \| \| The knight will *scramble/tiptoe* along the path to the fortress. \| \| The soldier will *sprint/creep* along the path to the castle. \| \| The businessman will *scramble/saunter* along the path to the fountain. \| \| The bodybuilder will *shoot/lumber* along the path to the statue. \| \| The nurse will *charge/meander* along the road to the café. \| \| The fox will *bound/crawl* along the track to the wigwam. \| \| The hunter will *hurry/tiptoe* along the track to the cabin. \| \| The tribesman will *run/sneak* along the track to the hut. \| \| The farmer will *scamper/waddle* along the track to the haystack. \| \| The boy will *shoot/mope* along the trail to the cave. \| \| The gardener will *bound/crawl* along the track to the shed. \| \| The girl will *scamper/amble* along the path to the flowers. \| \| The policeman will *rush/dawdle* along the lane to the telephone. \| \| The taxi will *hurtle/inch* along the lane to the traffic light. \| \| The duck will *speed/drift* along the stream to the reeds. \| \| The deer will *bolt/limp* along the trail to the rocks. \| \| The bear will *dash/trudge* along the trail to the tent. \| \| The student will *run/stagger* along the trail to the picnic basket. \| \| The woman will *jet/waddle* along the avenue to the church. \| \| The butcher will *charge/creep* along the avenue to the cow. \| \| The clown will *fly/meander* along the avenue to the ball. \| |
